# Occult Breast Lobular Carcinoma with Numerous Circulating Tumor Cells in Peripheral Blood

**DOI:** 10.1155/2015/135684

**Published:** 2015-06-25

**Authors:** Kanako Ogura, Maki Amano, Toshiharu Matsumoto, Asumi Sakaguchi, Taijiro Kosaka, Toshiaki Kitabatake, Kuniaki Kojima

**Affiliations:** ^1^Department of Diagnostic Pathology, Juntendo University Nerima Hospital, Tokyo 177-8521, Japan; ^2^Department of Radiology, Juntendo University Nerima Hospital, Tokyo 177-8521, Japan; ^3^Department of Breast Surgery, Juntendo University Nerima Hospital, Tokyo 177-8521, Japan

## Abstract

We experienced a very rare case of occult breast lobular carcinoma with numerous circulating tumor cells in peripheral blood. The diagnosis was very difficult because there were no symptoms of breast cancer and the preceding chief complaints such as general fatigue and weight loss or abnormality of peripheral blood findings were suggestive of a hematological disease. We could make a correct diagnosis of this case by checking the findings of complete blood count and bone marrow biopsy at the same time using immunohistochemistry.

## 1. Introduction

Invasive lobular carcinoma (ILC) accounts for about 6% of breast cancer, which is the second most common type after invasive ductal carcinoma (IDC) [1, 2]. It is well known that the pattern of metastatic spread of ILC is different from that of IDC, so scrupulous attention to metastasis to unusual foci such as the gastrointestinal tract, uterus, ovary, and meninges is required [3].

There are some reports of breast cancers with bone marrow metastases. Kopp et al. analyzed 22 of such cases, in which 7 cases were ILC [4]. Seventeen cases were diagnosed by bone marrow histology and the other 5 cases were revealed because of leukoerythroblastic blood smear.

On the other hand, occult breast carcinoma accounts for 0.3 to 1% of breast cancer [5]. It is defined as breast carcinoma with an axillary lymph node metastasis as the first clinical manifestation of the disease. Recently, diagnostic imaging has dramatically improved, so it has been recommended for occult breast carcinoma to perform mammography repeatedly or magnetic resonance imaging (MRI). The prognosis of occult breast cancer seemed to be similar to that of usual and overt breast cancer with lymph node metastasis.

We experienced a unique case of occult invasive lobular carcinoma with identification of circulating tumor cells in peripheral blood count. There have been very few reports that tumor cells themselves were identified in peripheral blood count, even if secondary change such as leukoerythroblastosis in peripheral blood smear was shown.

## 2. Case Presentation

**Figure 1:**
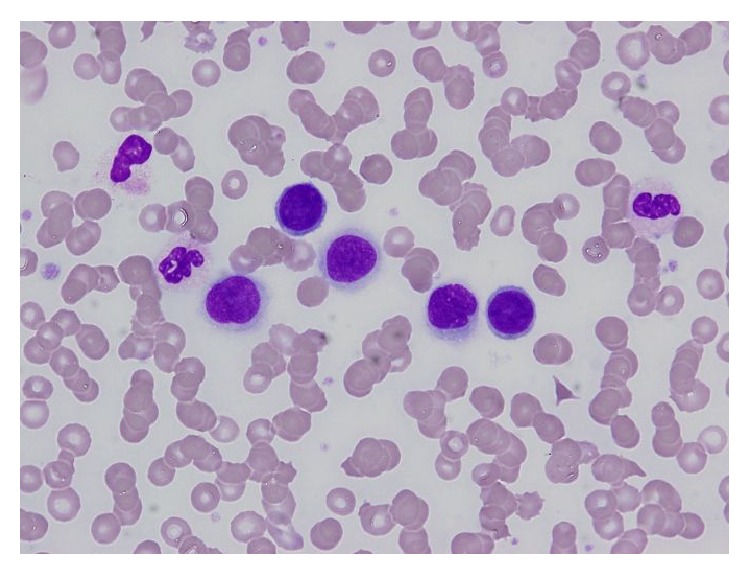
Tumor cells in peripheral blood smear. Noncohesive round tumor cells resembling lymphocytes were identified.

The patient was a 65-year-old woman, with chief complaints of general fatigue and weight loss. The findings of peripheral blood test are summarized in Table 1. The number of leukocytes was high (22,200/*μ*L) and atypical unclassified cells were identified in her peripheral blood count. Those cells were counted as “other” at a level of 7.5%. The atypical cells were noncohesive and had round nuclei and cytoplasm with microvilli resembling lymphocytes or plasma cells in form (Figure 1). The serum calcium level was high (13.4 mg/dL). Such findings leaded her physician into the suspicion of hematological disease such as lymphoma or myeloma although the serum level of CEA was also high (621 ng/mL).

**Figure 2:**
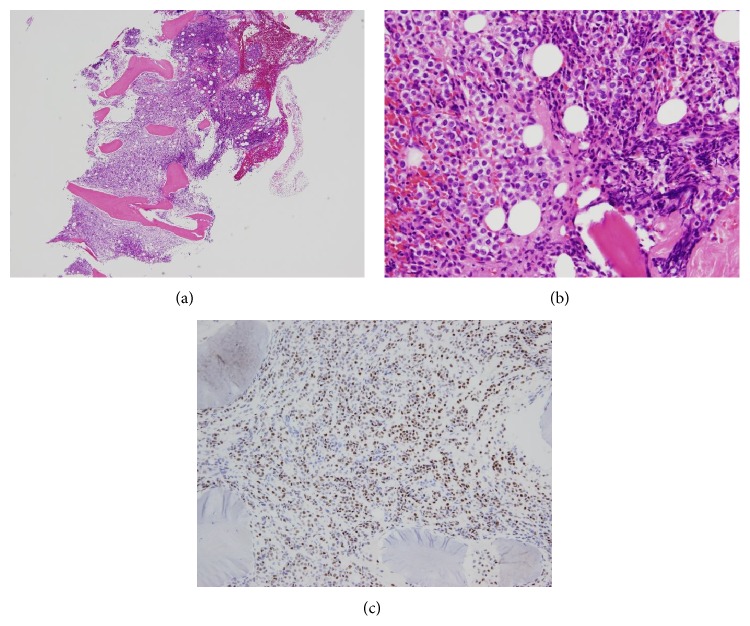
Bone marrow biopsy specimen. (a) H-E stain. Hypercellular bone marrow. (b) H-E stain. Monotonous tumor cells invaded bone marrow space accompanied by fibrosis. (c) Immunohistochemical stain of estrogen receptor. Tumor cells were diffusely positive.

**Figure 3:**
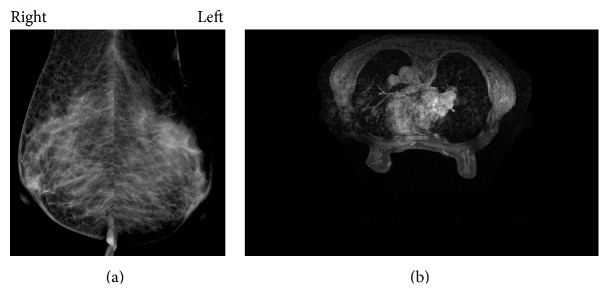
(a) Mammography. There was no lesion in either breast. (b) Enhanced MRI. There was also no lesion in either breast.

**Figure 4:**
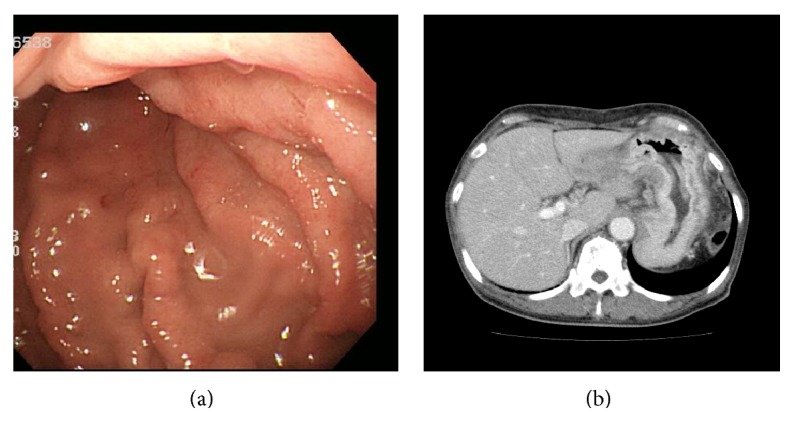
(a) Upper endoscopy. Mucosal rugae were edematous and thickened. (b) Contrast-enhanced CT revealed diffuse thickening of gastric wall.

**Figure 5:**
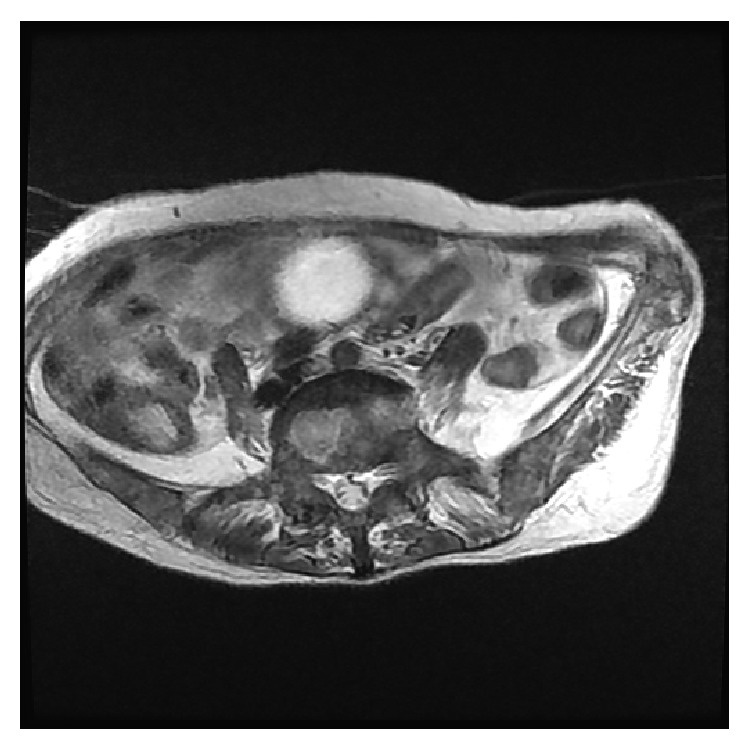
T1-weighted MRI revealed low-intensity lesion occupying lumbar spine and iliac bone diffusely.

**Figure 6:**
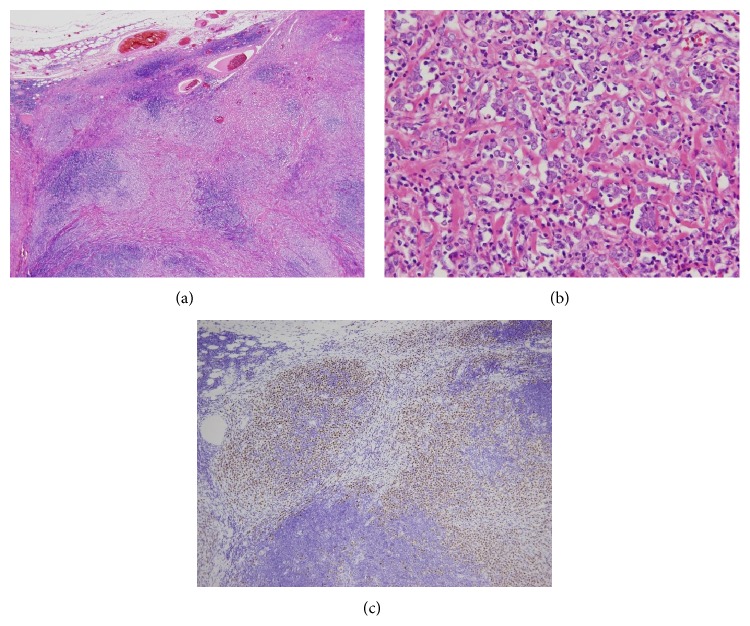
Axillary lymph node specimen. (a) H-E stain. Low power field. Tumor cells invaded lymph node parenchyma. (b) H-E stain. High power field. Monotonous tumor cells and fibrotic area. (c) Immunohistochemical stain of estrogen receptor. Tumor cells were diffusely positive.

**Figure 7:**
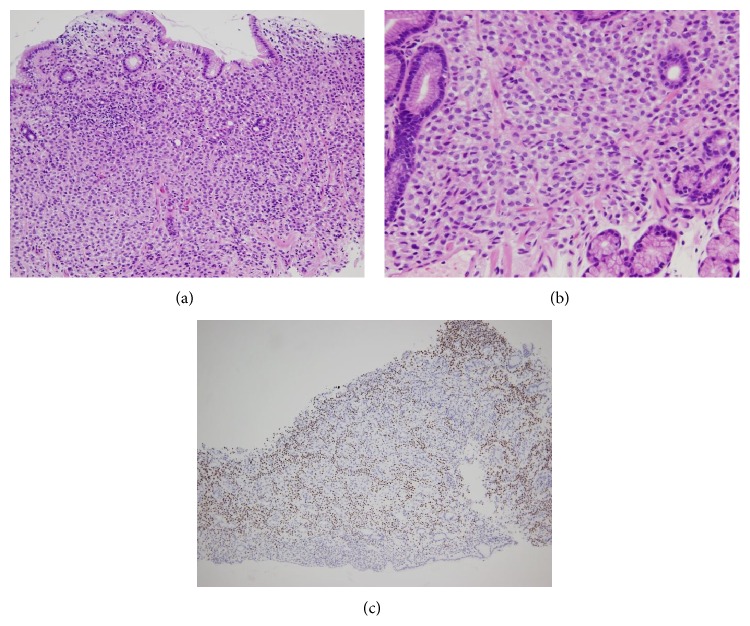
Gastric biopsy specimen. (a) H-E stain. Low power field. Tumor cells infiltrated the lamina propria. (b) H-E stain. High power field. Monotonous round tumor cells resembling the tumor cells seen in bone marrow and axillary lymph nodes. (c) Immunohistochemical stain of estrogen receptor. Tumor cells were diffusely positive.

Her physician performed a bone marrow puncture. The bone marrow specimen showed so-called “packed bone marrow” and fibrosis was also shown, so aspiration failed (so-called “dry tap”). In the biopsy specimen, numerous small round cells that were the same as the cells in the peripheral blood had grown with proliferation of reticular fibers (Figures 2(a) and 2(b)). We continued histological investigation using immunohistochemistry. These tumor cells were negative for LCA but positive for AE1/AE3. Furthermore, tumor cells were also positive for GCDFP15, ER, and (Figure 2(c)) PgR but negative for E-cadherin. On the basis of the result of immunohistochemistry, we diagnosed the bone marrow lesion as metastatic lobular carcinoma of the breast. Subsequently, the patient underwent detailed examination concentrating on imaging study. However, no lesion in either breast was identified by mammography (Figure 3(a)), ultrasonography, and even contrast-enhanced CT or MRI (Figure 3(b)). Enhanced CT images showed swelling of left axillary, right internal mammary, and perigastric lymph nodes and diffuse thickening of gastric wall like “Bormann 4 tumor” (Figure 4). T1-weighted MRI revealed generalized bone marrow metastasis. Fatty bone marrow was diffusely occupied by low-intensity lesion (Figure 5). Furthermore, bone scintigraphy also revealed generalized osseous metastasis by so-called “beautiful bone sign.” Biopsies from an enlarged left axillary lymph node and from gastric mucosa were performed, resulting in the finding of metastatic lobular carcinoma that we confirmed by immunohistochemistry including ER, PgR, and GCDFP15 (Figures 6 and 7). We summarized the immunohistochemical findings of bone marrow, axillary lymph node, and gastric biopsy specimens in Table 2. Carcinoma from accessory breast tissue in axilla was also ruled out.

Finally, this case was diagnosed as occult breast lobular carcinoma with multiple metastases to bone, bone marrow, left axillary lymph nodes, and stomach with many circulating tumor cells in peripheral blood.

After the diagnosis, combination chemotherapy with docetaxel, cyclophosphamide, and zoledronic acid hydrate was applied. During chemotherapy, diffuse thickening of upper dermis of the left breast appeared. Since the patient refused skin biopsy, the underlying cause of the skin swelling remains obscure. The symptoms such as swelling of axillary lymph nodes and thickening of dermis and gastric wall were improved by chemotherapy. Although enhanced CT has been repeatedly performed, there has been no lesion in either breast. At the time of writing the paper, the patient is still undergoing treatment.

## 3. Discussion

This case was difficult to diagnose because of an unusual clinical presentation, that is, identification of massive amount of nonhematological tumor cells in peripheral blood count and “occult” breast carcinoma.

Pentheroudakis et al. recommended performing mammography repeatedly or magnetic resonance imaging (MRI) for occult breast carcinoma [6]. They documented that primary small foci in both breasts were identified in 59% of cases of occult breast cancer that they reviewed by MRI imaging. They also revealed that a small breast primary focus was identified histologically in 72% of cases treated with mastectomy. However, primary foci were sometimes not identified. In our case, there was no lesion in either breast regardless of performing various modalities such as mammography, ultrasonography, CT, or MRI. Our case was thought of as true occult breast carcinoma. Neal et al. reported occult breast carcinoma (ILC) presenting as gastrointestinal metastases without lesions in the breasts [7]. Invasive lobular carcinoma has a tendency to metastasize to gastrointestinal tract. Metastatic breast lobular carcinoma can easily be mistaken for a primary gastrointestinal cancer. Metastatic “occult” lobular carcinoma is even more difficult to identify. They documented that immunohistochemistry could allow accurate diagnosis. In our case, tumor cells metastasized to stomach, axillary lymph node, and bone marrow. We could perform immunohistochemistry to all samples from stomach, lymph node, and bone marrow and compare the results. Immunohistochemistry was also powerful diagnostic tool in our case.

Such a situation when circulating tumor cells are visible in peripheral blood smear is extremely rare. In our case, hematological disease was suspected at the initial visit because of the many noncohesive tumor cells (7.5% of all blood cells) seen in the peripheral blood smear, suggesting hematological malignancy. There are reports of circulating tumor cells in primary breast cancer. However, the methods for the identification of circulating tumor cells are almost all molecular ones, such as RT-PCR. Fortunato et al. revealed that RT-PCR of the BM is an independent prognostic factor for disease-free survival of breast cancer patients [8]. This is the first case report of breast carcinoma with “countable” nonhematological tumor cells in a peripheral blood smear. In our case, bone marrow biopsy was also performed, so we could compare the circulating atypical cells in both peripheral blood smear and the biopsy sample with performing immunohistochemistry. It contributed to prompt and correct diagnosis of this difficult case.

Kopp et al. warned that bone marrow involvement has to be considered in breast cancer patients, in particular in those with bone metastases and otherwise unexplained cytopenia [4]. They also documented that full blood exam can serve as a simple diagnostic tool. Cotta et al. documented that lung, breast, and prostate cancers are prone to bone metastasis in adults [9]. If a carcinoma metastasises to bone marrow, leukoerythroblastic reaction can sometimes be found on full blood exam.

In our case, the patient was not suspected of having breast cancer at the first visit and bone marrow puncture proved to be the decisive tool in correct diagnosis. It may be necessary for breast cancer patients to perform not only complete blood count but also bone marrow puncture if some abnormalities on full blood exam are identified. Cotta et al. also documented that circulating tumor cells sometimes mimic the clinical appearance of acute leukemia. In cases of breast cancer, lobular carcinomas do metastasize to skeletal system in higher frequency. The tumor cells can be difficult to identify because they can be small and scant, with an appearance that is difficult to distinguish from that of lymphocytes or blast cells. Indeed, we also suspected lymphoma or plasmacytoma at first.

Lyda et al. documented that keratin immunohistochemistry (IHC) detected clinically significant metastases in bone marrow biopsy specimens in women with lobular breast carcinoma [10]. They divided the cases of ILC into three groups based on hematoxylin and eosin (H-E) staining and keratin IHC results. They showed that prognosis was worse in the order of H-E-negative/IHC-negative, H-E-negative/IHC-positive, and H-E-positive/IHC-positive. They concluded that routine morphologic examination without the aid of keratin IHC was unreliable in detecting clinically relevant metastatic lobular carcinoma in bone marrow biopsies. According to Lyda et al., our case was categorized into H-E positive/IHC positive case, so the patient's prognosis was to be expected as unfavorable. Immunohistochemistry in our case was not so much useful for prognostic prediction but helped in accurate diagnosis. We could reconsider the origin of the tumor cells by the immunohistochemical reactions: LCA was negative and ER, PR, and GCDFP15 were positive in the atypical cells in bone marrow, axillary lymph node, and gastric biopsy specimens. It was also important to check the findings of complete blood count and bone marrow biopsy specimen at the same time. Misdiagnosis or delay of diagnosis might have occurred if the diagnosis based on complete blood count preceded bone marrow biopsy.

In conclusion, we experienced a case of occult breast lobular carcinoma with numerous circulating tumor cells in peripheral blood count. Although the diagnosis was difficult because circulating tumor cells were abundant and mimicked lymphocytes or blast cells, immunohistochemistry was very useful for the correct diagnosis. We could also make a diagnosis quickly because we checked the patient's peripheral blood smear and bone marrow biopsy specimen at the same time.

## Figures and Tables

**Table 1:** The patient's peripheral blood data.

WBC	22200	/*μ*L
Neutrophil	64.5	%
Lymphocyte	20	%
Monocyte	7.5	%
Eosinophil	0	%
Basophil	0	%
Other	7.5	%
Myelocyte	0.5	%
Hb	13.7	g/dL
Ht	42.4	%
Plt	315 × 10^4^	/*μ*L

TP	8.2	g/dL
Alb	3.4	g/dL
AST	57	IU/L
ALT	20	IU/L
LDH	373	IU/L
ALP	973	IU/L
BUN	29	mg/dL
Cre	1.2	mg/dL
Na	135	mEq/L
K	4.9	mEq/L
Cl	94	mEq/L
Ca	13.4	mg/dL

CEA	621	ng/mL

**Table 2:** Immunohistochemical findings of this case.

	Bone marrow	Axial lymph node	Stomach
AE1/AE3	+	+	+
ER	+	+	+
PR	+	+	+
HER2	Score 1+	Score 1+	Score 1+
Mammaglobin	−	−	−
GCDFP15	+	+	+
CK7	+	+	+
CK20	−	−	−
CDX2	−	−	−
